# A VIRTUAL STAFF TRAINING TO IMPROVE COMMUNICATION FOR OLDER ADULTS DURING MASK WEARING

**DOI:** 10.1093/geroni/igac059.2023

**Published:** 2022-12-20

**Authors:** Sara Mamo, Katherine Garrity, Kara Wheeler

**Affiliations:** University of Massachusetts Amherst, Amherst, Massachusetts, United States; University of Massachusetts Amherst, Amherst, Massachusetts, United States; University of Massachusetts Amherst, Amherst, Massachusetts, United States

## Abstract

The need for masking protocols in care centers for older adults exacerbated communication challenges in an already difficult environment. The purpose of this study was to alleviate the burden of communicating while wearing masks by providing a virtual staff training that introduced a headset amplifier and communication tips. The training was delivered via Zoom to small groups of staff at three Programs for All-inclusive Care for the Elderly (PACE®) organizations. The training included education about the impact of age-related hearing loss, instructions for using a headset amplifier, and communication tips. Staff were encouraged to use the amplifier with as many participants as possible to ease communication while wearing masks—rather than targeting participants based on hearing status. A pre/post quasi-experimental approach was undertaken. Fifty-one staff members completed the training and immediate pre/post questionnaires to measure knowledge gain. Follow-up questionnaires (including open-ended responses) were collected at 2- (n = 29), 4- (n = 23), and 6-months (n = 23) post-training. In addition, we completed one focus group (n = 5) and one in-depth interview regarding the feasibility of participation in the research project and brainstorming to increase use of the amplifiers. By integrating quantitative and qualitative findings, we highlight communication improvements when using the amplifiers and tips for integrating amplifiers into group care programs. The findings from this study will contribute to the development of a large-scale intervention to address hearing loss and support communication for older adults in group care settings.

